# Ultrasonic replacement of natural aging: Potential strategies for improving the color, antioxidant activity, and volatile compound profile of astragalus mead

**DOI:** 10.1016/j.ultsonch.2025.107319

**Published:** 2025-03-20

**Authors:** Jianfeng Wang, Xiangjin Kong, Yuqi Han, Faisal Eudes Sam, Jixin Li, Zhengmei Qi, Yumei Jiang

**Affiliations:** a College of Food Science and Engineering, Gansu Agricultural University, Lanzhou 730070, China; b Key Laboratory of Geriatric Nutrition and Health, Ministry of Education, Beijing Technology & Business University, Beijing 100048, China; c College of Enology, Northwest A&F University, Yangling, Shaanxi 712100, China; d Gansu Runfengyuan Agricultural and Animal Husbandry Ecological Technology Co., Ltd., Yongdeng, Gansu 730300, China

**Keywords:** Astragalus mead, Non-thermal processing, Flavor, Ultrasonic, Aging

## Abstract

The growing demand for natural and functional beverages has driven research aimed at improving the quality of herbal meads. This study investigates the use of non-thermal processing methods, ultrasonic, microwave, and high hydrostatic pressure processing, as alternatives to traditional natural aging for improving the physicochemical properties, antioxidant activity, color stability, and volatile compound profile of astragalus mead. Response surface methodology was employed to optimize fermentation conditions, which yielded the highest flavonoid content and sensory quality at an impregnation time of 12 h, an impregnation temperature of 10 °C, and a fermentation temperature of 20 °C. Among the processing methods evaluated (natural aging, ultrasound, microwave irradiation, and high hydrostatic pressure), ultrasound treatment resulted in the most significant improvements. Specifically, it increased total phenol content by 7.22 %, total flavonoid content by 9.41 %, and antioxidant capacity by 65.43 %. Volatile compound analysis also revealed a 191.30 % increase in ester content, significantly enhancing floral and fruity notes. Sensory analysis using quantitative descriptive analysis, partial least squares discriminant analysis, and weighted gene co-expression network analysis confirmed the efficacy of ultrasound, with ethyl caprylate identified as a key aroma contributor. These findings suggest that ultrasound is an effective non-thermal processing technique for improving the aging process and overall quality of astragalus mead. This study provides valuable insights for the industrial application of non-thermal processing technologies in astragalus mead production.

## Graphical abstract

**Figure:**
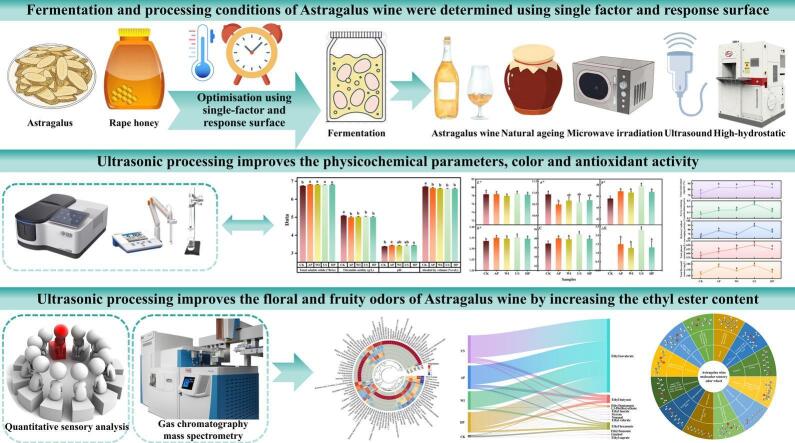

## 1 Introduction

*Astragalus membranaceus,* commonly known as 'huangqi', belongs to the largest genus of vascular plants, distributed in America and most of Eurasia [1]. *Astragalus membranaceus* contains a variety of active constituents, including astragalus polysaccharides, saponins, flavonoids, alkaloids, and amino acids [1]. These constituents have demonstrated numerous biological benefits, including enhanced immune function, anti-inflammatory and antioxidant properties, cardiovascular protection, anticancer effects, and improved metabolic health [2]. Specifically, astragalus has been shown to improve insulin resistance, regulate nerve growth factor, manage lipid metabolism, combat oxidative stress, influence apoptosis, and modulate the immune system, making it particularly useful for treating diabetic peripheral neuropathy [3]. Additionally, fermented astragalus has proven effective as a feed additive in poultry, improving egg quality and intestinal health [4]. Astragalus has also been incorporated into beverages, such as beer, to enhance immune function [5]. Honey processing of astragalus alters its polysaccharide structure, potentially enhancing its probiotic effects and short-chain fatty acid production [6]. Furthermore, combining honey with fruits, vegetables, or other herbs enables the creation of diverse beverage meads, enhancing both flavor complexity and health benefits [7]. Natural aging of fruit wines can enhance flavor by reducing undesirable freshness and spicinesswhile increasing softness and mellowness [8], [9]. During the aging process, various physical and chemical reactions continue until dynamic equilibrium is achieved [10]. Moreover, natural aging of fruit wines enhances flavor and texture through slow oxidation and complex chemical reactions. However, it is time-consuming and susceptible to microbial contamination and the risk of over-oxidation. Accelerated artificial aging techniques can significantly reduce aging time by controlling factors such as temperature, pressure, and catalysts, thereby improving production efficiency and consistency. In this regard, non-thermal processing technologies such as microwave irradiation, ultrasound, and high hydrostatic pressure have emerged as promising alternatives [11]. These methods not only reduce aging time but also accelerate esterification, enhance flavor release, and prevent microbial contamination [12]. Furthermore, they allow for precise control of physicochemical properties and organoleptic qualities, ensuring consistent product quality [13].

Among these technologies, high hydrostatic pressure has shown promising applications in food processing, sterilization, and quality improvement [14]. For instance, a comparison of high hydrostatic pressure-treated red wines with barrel-aged and micro-oxygenated wines showed that high hydrostatic pressure altered the phenolic composition of the wines, reducing the content of anthocyanins, phenolic acids, and flavonols, while promoting compound condensation reactions [15]. Ultrasonic treatment has been reported to enhance the volatile compound profile and overall quality of fruit wines by increasing the concentration of free varietal compounds like terpenes and norisoprenoids in musts and wines [16]. In cashew-apple juice, ultrasound processing reduced undesirable fatty acids and styrene while increasing fruity short-chain esters [17]. In fortified sweet wines, ultrasonic treatment during fermentation enhanced anthocyanin content, color stability, and aroma complexity [18]. Additionally, in steeped greengage wine, low-frequency ultrasound accelerated maturation by reducing fusel oils and alcohol compounds while increasing acid and ester compounds [19]. Across these studies, ultrasonic treatment generally improved the sensory attributes and aroma quality of the wines. However, the effects varied depending on the frequency, power, and duration of the ultrasonic treatment, as well as the specific type of fruit wine being processed. When the effects of ultrasound amplitude and time on the stability indices of anthocyanins and phenols were analyzed for two red wines, it was found that ultrasound aging favored the preservation of polyphenolic compounds and did not induce degradation reactions [20]. Microwave treatment, meanwhile, has been shown to reduce the content of higher alcohols in wine by up to 11.21 % under optimized conditions, potentially enhancing its aromatic profile [21]. During storage, microwave-treated red wines showed similar trends in color and phenolic compound changes to untreated wines, but at a faster rate, indicating its effectiveness as an accelerated aging method [22]. In the context of red winemaking, microwave-assisted maceration of crushed grapes influences yeast populations, enhances fermentation kinetics, and increases the extraction of amino acids and polysaccharides, particularly those rich in arabinose and galactose [23].

Despite the potential of non-thermal processing techniques, there is limited research comparing their effects on different types of fruit wines aimed at providing guidance for large-scale production. While much of the existing work focuses on raw material types, fermentation optimization, and strain selection, less attention has been given to comparing the impacts of different artificial aging methods. Additionally, mixed fermentation, involving multiple food raw materials, offers a way to overcome the limitations of single ingredient fermentation by enhancing flavor complexity, body, and nutritional profile. This approach can also allow the advantages of different raw materials to complement and enhance one another, significantly improving flavor and texture while providing complex aromas and more nutrients. Astragalus and rape honey offer unique advantages as food ingredients for mead production and other applications.

Therefore, this study aims to fill existing knowledge gaps in understanding the fermentation and aging of astragalus mead and provide insights into the potential of non-thermal processing techniques for accelerated aging. To this purpose, the fermentation conditions for astragalus mead were first compared using single-factor and response surface methodology (RSM). Subsequently, we compared the effects of different non-thermal processing methods, particularly natural aging, microwave irradiation, ultrasound, and high hydrostatic pressure, on the physicochemical parameters, color, antioxidant activity, and volatile compound profile of the mead. Finally, the sensory characteristics were evaluated using quantitative descriptive analysis (QDA). The findings of this research will promote the added value of agro-processing and further the development of astragalus and rape honey as ingredients in functional beverages.

## 2 Materials and methods

### 2.1 Raw materials and chemical reagents

Dried astragalus (*Astragalus mongholicus* Bunge) tablets were sourced from the leading local supplier (Runfengyuan Co., Ltd., Gansu, China), meeting Chinese national quality standards. The astragaloside IV and moisture contents were 0.053 % and 5.5 %, respectively. Rape honey was collected from the Chengdu Plain in Sichuan Province, with a reducing sugar content of 751.00 ± 41.0 g/L and pH 3.97 ± 0.01. Pectinase, cellulase, and rutin standards (purity ≥ 98 %) were purchased from Shanghai Yuanye Biotechnology Co., Ltd. Commercial *Saccharomyces cerevisiae* Aroma White was supplied by Enartis (45–110-0500, San Marino, Italy).

### 2.2 Laboratory-scale fermentation of astragalus mead

Fermentation tanks (5 L) were sterilized and decontaminated before use. Rape honey was diluted with water, thoroughly mixed, sterilized in an 80 °C water bath for 20 min, and then cooled to 25 °C. The dried astragalus tablets is crushed into a 200-mesh powder. A fermentation mash was prepared according to the following proportions: 80.6 % water, 16.9 % rape honey, and 2.5 % astragalus powder (*v/v/w*). Fermentation was initiated after maceration according to the parameters selected using single factor and response surface screening. *Saccharomyces cerevisiae* was added to the fermentation mash at 38 ± 0.1 °C after 30 min of activation, at a dosage of 0.3 g/L. Fermentation was carried out at a controlled temperature at 25 ± 2 °C. Fermentation kinetics were monitored by measuring alcohol content, and fermentation was stopped when the alcohol level fell below 7 % vol. The mixture was centrifuged at 10,000 rpm for 30 min and then filtered to obtain the fermented astragalus mead.

### 2.3 Single factor experiments and RSM optimization

The impregnation time (8, 12, 16, 20, and 24 h), impregnation temperature (5, 10, 15, 20, and 25 °C), and fermentation temperature (15, 18, 21, 24, and 27 °C) were evaluated to obtain the best fermentation conditions. The overall score was measured based on total flavonoid content (TFC, 40 %) and sensory evaluation score (60 %). After single factor experiments, a three-factor, three-level Box-Behnken design (Table S1) was used to analyze the relationship between fermentation conditions and the overall score (represented as Y). Data were analyzed using Design-Expert 13.0 software (Stat-Ease Inc., USA).

### 2.4 Sample preparation

Astragalus mead was prepared using the optimized fermentation parameters derived from single factor and RSM, serving as the control group (CK). Natural aging mead (AP) was produced by storing CK in a temperature-controlled cellar at 14 ± 4 °C and 61.0 ± 3.71 % relative humidity for six months. For microwave irradiation treated mead (WI), CK was irradiated at 640 W for 180 s (Scientz-II D, Xinzhi Co., Ltd., Ningbo, China). Ultrasound treated mead (US) was obtained by subjecting CK to ultrasound at 150 W for 600 s at 20 °C using the same Scientz-II D system. The ultrasound power, time, and temperature were determined from preliminary experiments conducted on the same batch of astragalus (Fig. S1). High hydrostatic pressure treated mead (HP) was produced by exposing CK to 400 MPa for 18 min using a high hydrostatic pressure machine (L2-600/1, Huataisenmiao Co., Ltd., Tianjin, China).

### 2.5 Sensory evaluation

Twenty-five candidate assessors aged 21–45 years (average age: 29) were selected from the Gansu Provincial Wine Technology Research and Development Centre, following the procedure outlined by Yu et al. [24]. Candidates signed an informed consent form and were free to withdraw from the training at any time. Ethical approval for the involvement of human subjects was granted by the Gansu Agricultural University Research Ethics Committee (GSAU-Eth-FSE-2023–012). The candidates, with over two years of sensory evaluation experience for fruit wine, Huangjiu, and Baijiu, were tested for sensory abilities, sensitivity, and descriptive skills. After evaluation, 21 candidates (8 males, 13 females) were selected as panel members. Subsequently, the panel underwent professional sensory training, following the method of Lawless et al. [25]. Training was conducted 4 times per week for 2.5 h for 4 weeks, covering sensory analysis basics, descriptor output, sensory characteristic identification, and scale development. During the main sensory evaluation, 30 mL of treated astragalus meads were presented in 50 mL special tasting cups, coded with three-digit random numbers. Panelists selected mead samples randomly for assessment. Scores were assigned according to the established evaluation criteria (Table S2), with coffee beans provided between samples to refresh the panel's sense of smell. Six evaluation dimensions for QDA (floral, fruity, fresh, woody, chemical, and vegetal odors) were selected according to Ng et al. [26]. Panelists used a 9-point scale, with 1 representing absent odor, 5 moderate intensity, and 9 extremely strong. The same panel was used for QDA to ensure consistency. For details on the definition of each QDA dimension, see Table S3.

### 2.6 Determination of physicochemical parameters

Titratable acidity was measured through acid-base titration [27]. The color parameters were determined using the procedure described by Han et al. [28]. Total soluble solids were determined with a PAL-1 pocket refractometer (Atago, Tokyo, Japan) and pH was measured using a pH meter (Inesa, Shanghai, China). Total phenol content (TPC) were determined by the Folin-Ciocalteu method, while total flavonoid content (TFC) was measured using the NaNO_2_-AlCl_3_ colorimetric method [29]. TPC and TFC were expressed as gallic acid and rutin equivalents, respectively.

### 2.7 Determination of antioxidant activity

Hydroxyl radical scavenging activity was evaluated following Bing et al. [30] with slight modifications. Briefly, 1 mL each of glutathione, astragalus mead, 2 mmol/L FeSO_4_·7H_2_O (1 mL), 6 mmol/L salicylic acid–ethanol solution (1 mL), and 6 mmol/L H_2_O_2_ (1 mL) were mixed. The mixtures were incubated at 37 °C for 30 min, and the absorbance was measured at 510 nm. Ferric reducing antioxidant power (FRAP) was evaluated following the method of Wu et al. [31]. Briefly, 200 μL of each mead was mixed with 1 mL 2,4,6-tripyridyl-*s*-triazine solution and incubated in a water bath at 37 °C for 20 min, followed by centrifugation at 10,000 rpm for 10 min. Absorbance was measured at 593 nm, and results were expressed as μg FeSO_4_/mL, using FeSO_4_ as the standard. Total antioxidant capacity was determined according to the kit instructions (S0119, Beyotime Co., Ltd., Shanghai, China).

### 2.8 Qualitative and quantitative analysis of volatile compounds

Referring to the method of Wang et al. [32] with slight modifications, 6 mL of astragalus mead was mixed with 1.0 g of NaCl and 30 μL of a combined internal standard (containing 10 μL of 59.64 μg/L of 2-octanol, 10 μL of 596.42 μg/L of ethyl 3-hydroxy-hexanoate and 10 μL of 19.88 μg/L of 1-octen-3-one). After sealing, the mixture was magnetically stirred at 40 rpm for 30 min. A solid-phase microextraction fiber (50/30 μm, DVB/CAR/PDMS, Supelco, United States) was then inserted, and headspace adsorption was carried out at a constant temperature of 40 °C and a stirring speed of 100 rpm for 30 min. Gas chromatography-mass spectrometry analyses were performed using a Thermo TRACE 1310-ISQ (Thermo Fisher Scientific, San Jose, CA, United States) equipped with a TG-WAX column (60 m × 2.5 mm × 0.25 μm). The temperature program was set to 40 °C for 5 min, then increased at a rate of 5 °C/min to 180 °C for an additional 10 min, with a carrier gas (He) flow rate of 1 mL/min. The electron energy was 70 eV, and the temperatures of the transmission line and ion source were 250 °C. The mass scanning range was *m*/*z* 50–450. A NIST spectral library search was combined with relative retention index characterization and internal standard quantification for the identification and quantification of volatiles. The odor activity values (OAV), defined as the ratio of the concentration of a volatile compound to its threshold in water, were calculated for each volatile compound. The detection thresholds of volatile compounds in water were obtained from a book titled ‘Compilations of odor threshold values in air, water and other media (Edition 2011)’ and Giri et al. [33]. The odor descriptors were obtained from two online databases (https://www.perflavory.com/, https://www.femaflavor.org/).

### 2.9 Statistical analysis

The means were compared using Tukey’s post hoc test, and differences at *p* < 0.05 were considered significant. Radar plots and bar plots were generated using OriginPro 2024 (OriginLab, MA, USA) and Graphpad Prism 9.5.1 (San Diego, CA, USA). Significance analysis and correlation analysis were performed using IBM SPSS Statistics 25 software (SPSS Inc., Chicago, IL). Partial least squares discriminant analysis (PLS-DA) was performed on MetaboAnalyst 6.0 using auto-scaling in normalization procedure (https://www.metaboanalyst.ca/). Random forest analysis and weighted gene co-expression network analysis of volatile compounds were performed using the RandomForest and WGCNA packages of R studio software, respectively.

## 3 Results

### 3.1 The single-factor optimization

**Fig. 1:**
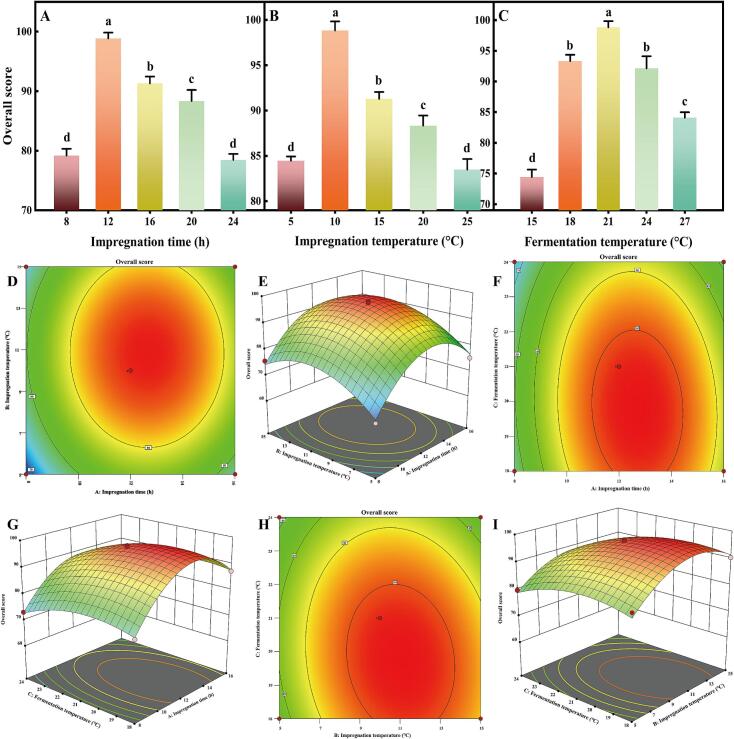
Optimization of fermentation conditions for astragalus mead using single-factor and response surface methodology. (A) Effect of impregnation time. (B) Effect of impregnation temperature. (C) Effect of fermentation temperature. (D) 2D contour plot and (E) 3D response surface of the interaction between impregnation time and impregnation temperature. (F) 2D contour plot and (G) 3D response surface of the interaction between impregnation time and fermentation temperature. (H) 2D contour plot and (I) 3D response surface of the interaction between impregnation temperature and fermentation temperature. Significant differences (*p* < 0.05) are indicated by different lowercase letters.

The overall score was calculated as the sum of 40 % TFC and a 60 % sensory evaluation score [34]. Initially, the score increased, peaking at an impregnation time of 12 h, where both TFC and sensory scores were maximized. Therefore, the optimal impregnation time range for response surface optimization was set at 8 to 16 h (Fig. 1A). As for impregnation temperature, the overall score followed a single-peak pattern, peaking at 10 °C. Higher temperatures reduced TFC and the overall score, leading to a chosen optimization range of 5 to 15 °C (Fig. 1B). Similarly, the overall score peaked at a fermentation temperature of 21 °C, with a slight decrease at 24 °C, establishing the optimization range of 18 to 24 °C (Fig. 1C).

### 3.2 The RSM optimization

Building on the outcomes of the single-factor experiments, we framed the RSM optimization. The Box-Behnken design matrix (Table S4), included three factors: (A) impregnation time, (B) impregnation temperature, and (C) fermentation temperature, each assessed at three levels along with the overall score. The optimal regression equation deduced from the analysis is as follows: *Y* = 96.42 + 4.10*A* + 2.89*B*-3.31*C*-0.21*AB*-1.00*AC*-1.38*BC*-12.86*A*^2^-8.38*B*^2^-4.38*C*^2^. Significance test for the regression coefficient and variance analysis showed *p*-values less than 0.0001 for the regression model and greater than 0.05 for the lack-of-fit term (Table S5). This indicates that the model is robust and has a good fitting degree. The ranking of F-values revealed that the factors contributing to the overall score were in the order: impregnation time (A) > fermentation temperature (C) > impregnation temperature (B). To further investigate the effects of these three factors and their interactions on the overall score, response surface and contour plots were created (Fig. 1D-I). The three-dimensional response surfaces of impregnation time and impregnation temperature displayed steep slopes, indicating a significant interaction between these two factors and their influence on overall score levels. In contrast, the two-dimensional contour plot, appeared approximately circular, suggesting a low level of interaction between the two factors. Similar observations were made regarding the interactions between impregnation time and fermentation temperature, as well as between impregnation temperature and fermentation temperature.

Finally, the optimized conditions for astragalus mead fermentation were established: an impregnation time of 12.80 h, an impregnation temperature of 10.49 °C, and a fermentation temperature of 19.60 °C. Under these conditions, the theoretical overall score reached 97.67. To enhance practicality and ease of implementation, these parameters were finetuned to an impregnation time of 12 h, an impregnation temperature of 10 °C, and a fermentation temperature of 20 °C. Under the adjusted optimized conditions, the overall score was 96.03, showing strong agreement with predicted values.

### 3.3 Effect of different treatments on the physicochemical parameters

**Fig. 2:**
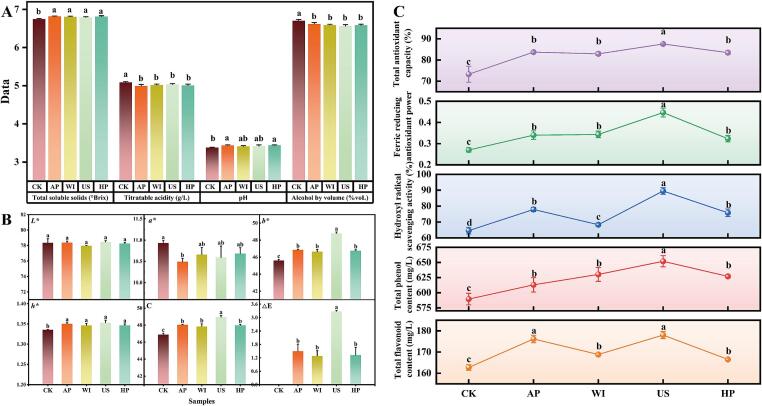
Influence of different non-thermal processing technologies on the quality of astragalus wine after aging. (A) Physicochemical parameters. (B) Color parameters. (C) Total phenolics, total flavonoids, and antioxidant activity.

Astragalus mead was fermented under the optimized conditions and the effects of different non-thermal processing technologies on the quality of astragalus mead were evaluated. For the treatment conditions involving microwave irradiation and high hydrostatic pressure, we referred to parameters established in previous studies [35], [36]. Due to the lack of literature on ultrasonic treatment, a single factor experiment was conducted for those conditions (Fig. S1). Fig. 2A presents the results regarding the physicochemical parameters of astragalus mead subjected to natural aging, microwave irradiation, ultrasound, and high hydrostatic pressure. The alcohol content of AP, HP, WI, and US treatments was significantly lower than that of CK. In addition, the titratable acidity of the four treated meads was significantly reduced compared to CK. Both AP and HP resulted in increased pH levels, with no significant difference among the treated meads. Interestingly, AP exhibited the lowest titratable acidity and the highest soluble solids content. In summary, the physicochemical parameters of astragalus mead were significantly altered by the different processing methods employed.

### 3.4 Effect of different treatments on the color parameters of astragalus mead

Fig. 2B shows the differences in color parameters among the various treatments of astragalus mead. The L* values of AP, WI, US, and HP meads ranged from 77.97 to 78.46, showing minimal variation and no significant difference from CK. The a* values for AP, WI, US, and HP meads were lower than those of CK, although a significant reduction was only observed between AP and CK. Importantly, the b* values for AP, WI, US and HP meads were significantly higher than CK, and there was no significant difference observed between natural aging and accelerated aging. Notably, the b* value of US improved significantly by 6.99 % compared to CK. Overall, the most pronounced color change was observed in the US mead, with a total color difference (ΔE) value of 3.28.

### 3.5 Effects of different treatments on the TPC, TFC, and antioxidant activity of astragalus mead

Fig. 2C shows the differences in TPC, TFC, and antioxidant activity among the different processing methods for mead. The TPC and TFC of AP, WI, US and HP treatments were significantly higher than that of CK. Notably, the ultrasonic treatment proved to be more effective, leading to increases in TFC and TPC of CK mead by 9.41 % and 7.22 %, respectively. For TPC, ultrasonic treatment improved the levels by 3.45 % compared to WI and by 3.97 % compared to HP. In summary, ultrasonic treatment was more beneficial than natural aging in facilitating the extraction and retention of phenolic compounds in astragalus mead. In addition, the FRAP, hydroxyl radical scavenging activity, and total antioxidant capacity of AP, WI, US, and HP meads were all significantly higher than CK. Specifically, FRAP of AP, WI, US, and HP meads increased by 25.93 %, 27.16 %, 65.43 %, and 19.75 %, respectively, compared to CK. Similarly, the hydroxyl radical scavenging activity increased by 20.66 %, 5.78 %, 38.64 %, and 17.36 %, respectively, compared to CK. The total antioxidant capacity also increased by 14.23 %, 13.14 %, 19.50 %, and 13.91 %, respectively, compared to CK. Compared with natural aging, ultrasonic treatment was more effective than WI and HP in improving the antioxidant activity of astragalus mead.

### 3.6 Effect of different treatments on the variety and concentration of volatile compounds

**Fig. 3:**
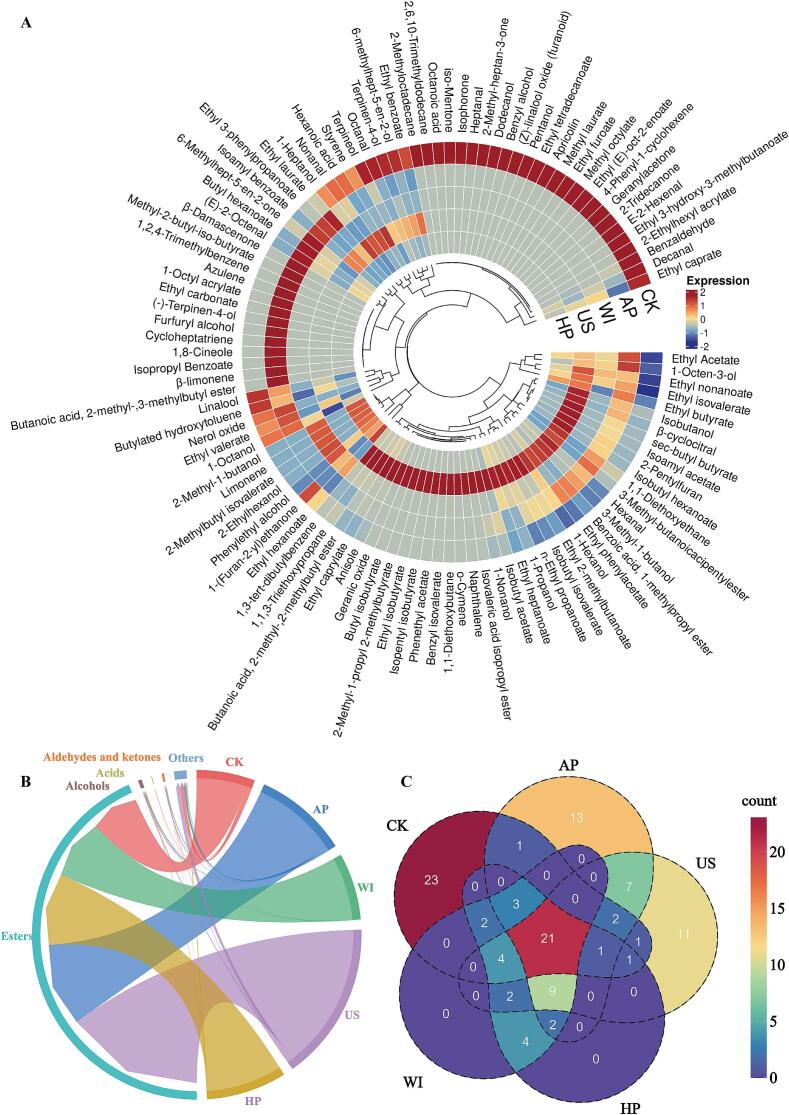
Impact of non-thermal processing treatments on volatile compound composition in astragalus mead. (A) Cluster heatmap of volatile compounds in different astragalus meads, normalized by the z-score method. (B) Composition and differences in volatile compound categories in different astragalus meads. (C) Venn diagram showing the types of volatile compounds in different astragalus wines.

A total of 104 volatile compounds were qualitatively and quantitatively identified in the five types of astragalus mead, including 43 esters, 21 alcohols, 2 acids, 16 aldehydes and ketones, and 22 others (Fig. 3A). The total volatile compound content, in descending order, was US > AP > HP > WI > CK. The total volatile compound content of US mead was 13,676.72 μg/L, an increase of 173.18 % compared to CK and 69.36 % compared to AP. Ethyl caprylate consistently ranked as the dominant compound across all meads, with concentrations ranging from 2,194.91 to 4,975.25 μg/L. Furthermore, all volatile compounds were divided into five subcategories (Fig. 3B). Esters formed the largest subcategory, accounting for 90.00 to 98.38 % of the total content, followed by “others” (0.63–6.91 %), alcohols (0.55–1.42 %), aldehydes and ketones (0.23–1.32 %), and acids (0–0.36 %). Notably, the ester content in US mead was significantly higher than in CK, AP, WI, and HP, at 191.30 % more than CK. Conversely, the alcohol content in US mead was significantly higher than CK by 40.94 %, while AP, WI, and HP had lower alcohol content compared to CK by 26.75 %, 21.48 %, and 62.40 % respectively. Acid content in AP, WI, US, and HP showed significant reductions of 100.00 %, 96.39 %, 55.41 %, and 97.13 %, respectively, compared to CK. Furthermore, the aldehyde and ketone content in AP, WI, US, and HP meads decreased significantly by 45.40 %, 76.73 %, 36.64 %, and 75.60 %, respectively, relative to CK. A Venn diagram was further used to illustrate the differences in volatile compounds among the astragalus meads (Fig. 3C). It revealed that all types shared 21 common volatile compounds, including 11 ethyl esters, 2 terpenes, and 2 straight-chain aldehydes (e.g., ethyl butyrate, linalool, and hexanal). CK contained 23 unique volatile compounds, including methyl octylate, 2-ethylhexyl acrylate, and heptanal.

### 3.7 PLS-DA and random forest analysis of volatile compounds

**Fig. 4:**
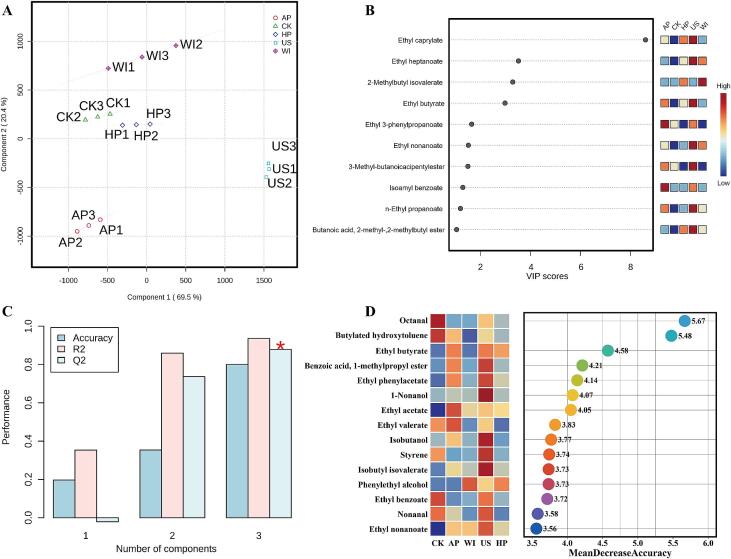
Partial least squares discriminant analysis (PLS-DA) and random forest analysis of volatile compound profiles in astragalus meads. (A) PLS-DA score plot of volatile compounds. (B) VIP value distribution. (C) 200 permutation test. (D) Biomarker distribution from random forest analysis.

To visually assess the overall differences in the volatile profiles of different astragalus meads and to identify the dominant compounds contributing to these differences, total volatile compound content was used as input data for PLS-DA and random forest analyses. The PLS-DA score plot shows a clear distinction among the five meads based on their score points (Fig. 4A). In particular, the US sample and the other four samples were positioned far apart on component 1. AP sample was clearly distinguished from CK, WI, and HP in component 2. In addition, the sum of the two components explained 89.90 % of the total variance. Using variable importance in projection (VIP) threshold greater than 1, 10 major compounds that significantly contributed to the differences among the samples were selected from a total of 104 volatile compounds (Fig. 4B). Notably, ethyl caprylate had the highest VIP value, reaching 8.60. However, aside from ethyl caprylate, ethyl heptanoate, 2-methylbutyl isovalerate, and ethyl butyrate, the VIP values were only between 1.0 and 2.0 for the other six dominant compounds. To evaluate the stability and fit of the PLS-DA model, a permutation test was conducted 200 times (Fig. 4C). The cumulative accuracy, Q^2^, and R^2^ of the three components all exceeded 0.8, indicating that the model for volatile compounds is stable and can effectively predict the composition and content of volatile compounds in astragalus mead. Finally, random forest analysis was utilized to identify biomarkers that distinguish astragalus mead from other fruit meads (Fig. 4D). Based on the mean decrease in accuracy, 15 volatile compounds were identified as potential biomarkers, with octanal and butylated hydroxytoluene serving as excellent indicators for detecting processing adulteration and sales fraud in astragalus mead.

### 3.8 Identification of odor-active compounds and QDA

**Fig. 5:**
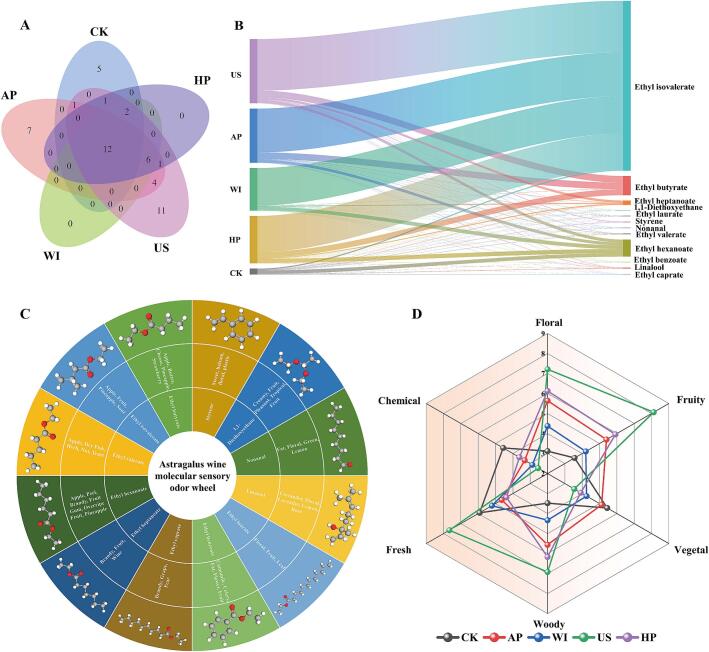
Screening of active odor compounds in astragalus mead, molecular sensory odor wheel construction, and quantitative descriptive analysis. (A) Venn diagram of odor-active compounds (OAV > 1) in different meads. (B) Sankey diagram showing aroma descriptors for 12 common active odor compounds. (C) Molecular sensory odor wheel with aroma descriptors and 3D structural formulas for these compounds. (D) Quantitative descriptive analysis of aroma profiles in different astragalus meads.

To determine the contribution and characterization of the odor-active compounds to the overall aroma profile of astragalus mead, OAVs were calculated from the odor thresholds in water and the compound concentrations. The active odor compounds in each type of astragalus mead were identified using an OAV threshold greater than 1. CK, AP, WI, US, and HP contained 21, 31, 20, 38, and 22 odor-active compounds, respectively. Twelve common active odor compounds were identified across all astragalus meads using a Venn diagram (Fig. 5A). CK, AP, and US each contained 5, 7, and 11 unique odor-active compounds, respectively, which may contribute to the characteristic aroma profiles of each mead. The distribution of common odor-active compounds with OAV values > 1 in different astragalus meads is illustrated in a Sankey diagram (Fig. 5B). All aged astragalus meads (WI, AP, US, and HP) showed significantly higher OAV values for common odor-active compounds than the control (CK), indicating that both natural aging and non-thermal processing enhance the flavor profile of astragalus mead. Among these, the OAV of ethyl isovalerate was highest in the ultrasonic-processed mead (US). A molecular sensory odor wheel was created by combining aroma descriptors of 12 odor-active compounds with OAV values > 1 and their structural formulas (Fig. 5C). The mead was primarily characterized by floral, fruity, and vegetal aromas. This wheel offers insights for product development, sensory evaluation, and processing specifications. Although instrumental analysis highlighted the significant effects of non-thermal processing, particularly ultrasonic treatment, further sensory evaluations by a professional panel are needed for consumer insights. Quantitative Descriptive Analysis (QDA) across six dimensions confirmed that the mead's aroma was predominantly floral and fruity, consistent with the OAV-based odor wheel (Fig. 5C). AP, WI, US, and HP significantly enhanced floral, fruity, and woody notes while reducing chemical odors. In particular, ultrasonic processing (US) resulted in a 131.04 % significant increase in floral, 128.23 % in fruity, and 98.75 % in woody notes compared to CK. No significant differences were observed among WI, US, and HP for the vegetal note, although all treatments significantly reduced it compared to CK and AP. The chemical odor was significantly reduced in AP, WI, US, and HP by 27.19 %, 36.95 %, 44.26 %, and 20.50 %, respectively, compared to CK.

### 3.9 WGCNA of sensory characteristics and volatile compounds

**Fig. 6:**
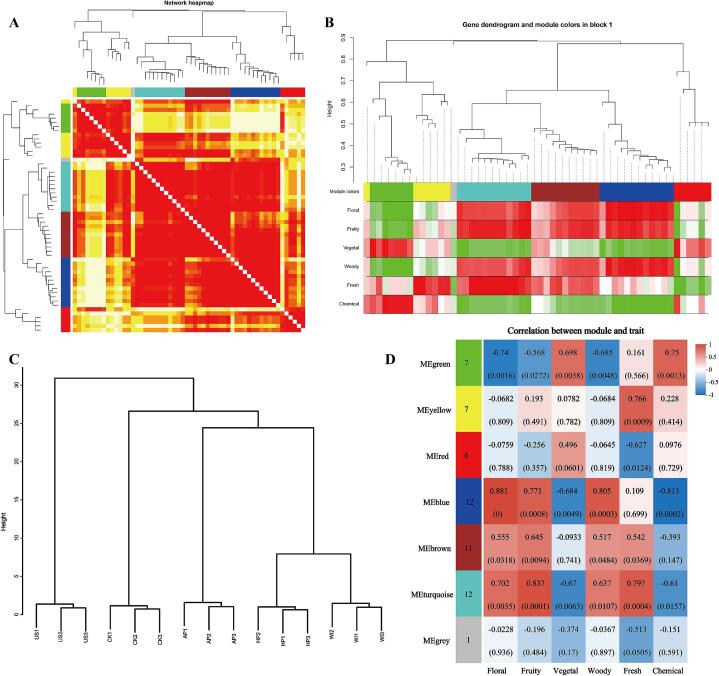
Identification of key compounds associated with floral and fruity notes using weighted gene co-expression network analysis (WGCNA) of volatile compounds. (A) Cluster and co-expression network heatmap of volatile compounds. (B) Color-coded module division and their association with sensory characteristics. (C) Cluster analysis of volatile profiles in different astragalus meads. (D) Correlation analysis of color-coded modules with sensory characteristics.

To further correlate sensory characteristics with volatile compounds, a WGCNA was constructed following the procedure used for black tea volatile compounds [37]. A soft threshold power of 6 was selected based on the scale-free topology fitting index and average connectivity (Fig. S2). The similarity of volatile compound expression was calculated from the expression value matrix, generating an adjacency matrix, which was then used to compute a topological overlap matrix. Hierarchical clustering of highly co-expressed lipids generated a cluster tree, from which six distinct modules, each represented by a unique color, were identified (Fig. 6A). Volatile compounds within each cluster exhibited high co-expression similarity, whereas similarity between clusters was low. A heatmap was generated to visualize the expression levels of co-expressed volatile compounds across seven modules, facilitating further module screening. Color gradients represented varying correlation coefficients among volatile compounds, with darker blocks indicating higher correlations. Compounds within the same module exhibited strong co-expression, while pronounced differences existed between modules (Fig. 6B). The color-coded modules were linked to sensory characteristics for further analysis. Cluster analysis of volatile compound profiles revealed that the US treatment was the most distinct, while AP, WI, and HP showed similar profiles, and CK formed a separate group. This aligns with the PLS-DA score plot results (Fig. 4A), indicating that ultrasonic treatment most effectively improved the flavor quality of astragalus mead, whereas AP, WI, and HP had similar efficacy. Correlation analysis of the six sensory characteristics and the seven color-coded modules identified those modules highly positively correlated with favorable sensory traits (Fig. 6D). The blue, brown, and turquoise modules showed strong correlations, with the turquoise module exhibiting the highest correlation coefficients with floral, fruity, and woody aromas, along with the smallest p-value. This suggests that the 12 volatile compounds in the turquoise module may be key contributors to these aromatic changes.

## 4 Discussion

### 4.1 Optimal fermentation conditions for astragalus mead using single factor and RSM

**Fig. 7:**
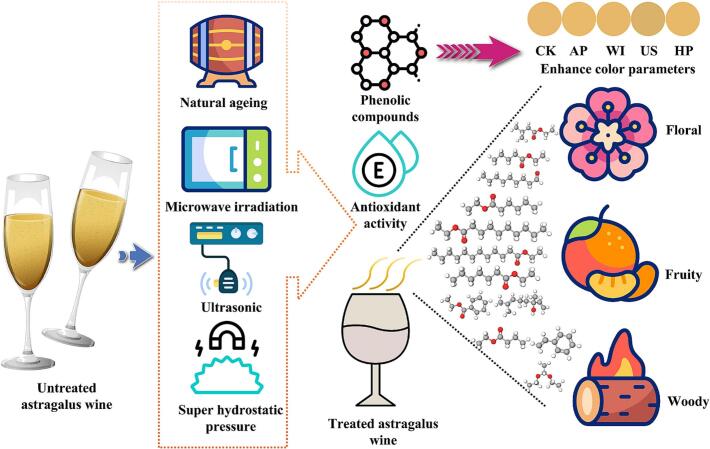
Comparative model of ultrasound and other non-thermal processing techniques on astragalus mead. Color round cards represent color parameters obtained from the online resource (https://www.colortell.com/). Icons are sourced from the premium version of an online resource library (https://www.freepik.com/).

Previous studies have demonstrated the effects of various non-thermal processing techniques on the physicochemical properties, antioxidant capacity, color attributes, and aromatic profiles of fermented beverages. For instance, ultrasonic treatment has been shown to increase phenolic content and enhance aroma complexity in fruit wines by increasing the concentration of key volatile compounds [16], [38]. Similarly, high pressure processing has been effective in improving color stability and boosting antioxidant activity in jujube wines [39]. Microwave treatment has been reported to significantly enhance phenolic extraction and influence aroma profiles in red wines, improving fermentation kinetics and increasing levels of volatile compound levels, which enhances red fruit and floral notes, even in sulfite-free wines [40]. Furthermore, high pressure processing preserves bioactive compounds in fruit-based beverages while modifying aroma profiles, reducing undesirable volatiles, and boosting fruity and floral notes, which are often affected by traditional thermal processes [41]. Natural aging, on the other hand, alters volatile compound concentrations through slow oxidative processes. While it can reduce primary aromas and develop tertiary notes, adding complexity to the wine, it also carries the risk of flavor degradation if not well-controlled [42]. However, research on herbal-fermented beverages, such as astragalus mead, remains limited. In this study, single factor and RSM were employed to optimize fermentation parameters, achieving significant improvements in TFC and sensory quality. This study also provides a unique comparison of the effects of aging, microwave, ultrasound, and high hydrostatic pressure treatments on the volatile and antioxidant profiles of astragalus mead. Ultrasonic processing, in particular, was found to significantly increase phenolic content and antioxidant capacity, and enhance floral and fruity aromas. The differences in the impact of these various non-thermal processing techniques on astragalus mead quality were comprehensively evaluated through the development of functional models (Fig. 7). These findings contribute to a better understanding of non-thermal processing and its role in optimizing the sensory and nutritional qualities of herbal-fermented beverages, offering potential strategies for industrial applications.

Recent studies have also explored optimizing fermentation parameters for fruit wines and meads using single factor and RSM approaches. Key factors investigated include temperature, pH, sugar content, yeast concentration, and fermentation time. For sour cherry spirit production, optimal conditions were found to be 24.71 °C, pH 3.25, and 22.49 °Brix, yielding 9.02 % *v/v* alcohol [43]. This contrasts with the optimal fermentation temperature of 20 °C for astragalus mead in this study, possibly due to differences in the yeast strains and fermentation mash. In mead production, initial dry matter content significantly affected alcohol yield and fermentation rate, with optimal conditions of 24.4 % dry matter, 150 mg/L yeast, and 140 mg/L yeast energizer [44]. The yeast additions were similar to those used in this study. Challenges in mead fermentation, such as delayed or arrested fermentations and undesirable sensory qualities, have been mitigated through nutrient additions and technological advancements [45]. For Thai white rice alcohol production, RSM was used to optimize the yeast-to-sugar ratio, sugar concentration, and fermentation time, yielding the best results at 4.0 mL yeast per 100 mL sugar solution, 20 °Brix, and 7 days fermentation period [46]. Fermentation time, an important factor affecting the organoleptic quality of fruit wines, was similarly applied in this study, as aligning closely with that of Thai white rice wine.

### 4.2 Ultrasonic processing enhances physicochemical properties and color of astragalus mead

Recent studies have shown that ultrasound treatment can significantly enhance the physicochemical properties and color parameters of fruit wines. It accelerates wine maturation, improves fermentation, and enhances color stability by increasing anthocyanin content and reducing degradation [47], [48]. Therefore, it was speculated that the 150 W ultrasonic treatment improved color performance in astragalus mead by enhancing flavanol content and reducing degradation. This technology improves a*, b*, and C* values, as well as color intensity, which is related to copigmentation [48]. Additionally, ultrasound treatment enhances antioxidant properties, modifies rheological behavior, and improves the sensory attributes of fruit juices [49]. The mechanism behind these improvements involves the accelerated polymerization of flavan-3-ols bridged by acetaldehyde and glyoxylic acid, following a first-order reaction model that is non-spontaneous and endothermic [50]. Although previous studies demonstrated that neither ultrasonic bath nor probe experiments caused significant changes in the color properties of wine [51], these findings suggest that ultrasound technology is a promising method for enhancing the physicochemical parameters and color quality of astragalus mead.

### 4.3 Ultrasonic treatment enhances antioxidant activity by modifying phenol retention and extraction

Recent studies have explored the effects of ultrasound treatment on alcoholic beverages and fruit extracts. Ultrasound technology has been shown to enhance wine maturation, improve fermentation, accelerate aging, and increase anthocyanin content by up to 50 % in red wine [47]. In astragalus stems and leaves, ultrasound-assisted extraction optimized the yield of flavonoids, especially isoquercitrin and astragalin, enhancing antioxidant activity [52]. This is in general agreement with the present study’s findings, which reported increased TPC and TFC. For Sanhua plum wine, ultrasonic treatment at 28 and 40 kHz enhanced color stability and antioxidative ability by inactivating polyphenol oxidase and reducing anthocyanin degradation [48]. This suggests that ultrasound treatment effectively retains phenolic compounds and enhances antioxidant activity, regardless of variations in power, duration, and food ingredients. In peach beverages, ultrasound processing showed greater retention of TPC and improved free radical scavenging activities compared to pasteurization and microwave treatments during storage [53]. These findings highlight the superiority and versatility of ultrasound treatment as a non-thermal processing technique. Overall, ultrasound technology can significantly enhance phenolic content and antioxidant activity in various fruit-based alcoholic beverages and extracts, likely due to its cavitation effect and ability to disrupt cell membranes.

### 4.4 Ultrasonic enhances floral and fruity notes by increasing the ethyl ester content

Recent studies have explored various techniques to enhance the quality and flavor of alcoholic beverages. Ultrasound treatment has shown promise in improving wine maturation, fermentation, and aging processes while enhancing phytochemical, physicochemical, and organoleptic properties [47]. This study adds to the body of research on ultrasound's effects on sensory properties and volatile compounds. In white wine, ultrasound processing modified phenolic and volatile compositions, offering the potential for producing low-sulfite wines [51]. Notably, ultrasonic bath experiments indicated that higher bath temperatures led to the degradation of volatile compounds, especially esters and higher alcohols. This finding contrasts with our results, where ultrasound increased the content of ethyl esters in astragalus mead. This is possibly due to differences in sonication methods between the water bath and probe types. Similarly, low-frequency ultrasonic treatment significantly improved the flavor profile of watermelon juice by increasing volatile compounds and enhancing sweet, floral, and fruity notes [54]. The findings from watermelon juice corroborate the increase in floral and fruity aromas observed in this study. In Chinese rice wine, a combination of micro-oxygenation and electric field treatment effectively accelerated aging and improved sensory characteristics, yielding results comparable to natural aging [55]. This aligns with our study's findings that ultrasonic treatment produced higher quality results than microwave radiation, high hydrostatic pressure, and natural aging of astragalus mead. Collectively, these studies highlight the potential of non-thermal technologies to enhance the quality and flavor of various beverages, offering alternatives to traditional aging methods while potentially reducing production time and costs.

Extensive studies have examined the effects of ultrasound treatment on alcoholic beverages, particularly regarding volatile compounds and flavor enhancement. Ultrasound accelerates aging, improves fermentation efficiency, and enhances the overall quality of wines and spirits [56], [57]. In Chinese rice wine, ultrasound treatment increased ethanol yield by 23.53 % and significantly boosted ester content, with some compounds increasing by over 100 % [57]. This agrees with the substantial increase in ethyl ester volatile compounds observed in US mead. Similarly, potato wine treated with ultrasound showed increased ester content and improved sensory attributes, such as fruitiness and sweetness [58]. These enhancements are attributed to ultrasound's cavitation effect, which leads to rapid bubble formation and collapse, disrupting cell membranes and enhancing the extraction of flavor compounds [59].

### 4.5 *Research limitations and future work*

This study provides a strong foundation for understanding and optimizing the quality of astragalus mead through non-thermal processing, yet several limitations warrant further research. While optimized impregnation and fermentation conditions were identified, sensory evaluation relied on overall scores without detailed consumer feedback. Future studies should incorporate large-scale sensory testing for broader validation. The efficacy of ultrasound (US) in enhancing phenolic content, antioxidant activity, and flavor was demonstrated, but key US parameters (e.g., frequency, amplitude, power) remain unexplored. Investigating these variables could refine processing conditions and improve quality. Additionally, the economic feasibility, energy efficiency, and environmental impact of non-thermal treatments (e.g., WI and HP) were not assessed, necessitating life-cycle analyses for industrial applications. Beyond physicochemical and volatile analyses, the impact of processing on the bioavailability of bioactive compounds should be explored to enhance the nutritional relevance of treated meads. Furthermore, while WGCNA identified volatile compound modules linked to sensory attributes, validation using advanced machine learning models and gas chromatography–olfactometry is needed to improve predictive accuracy. The regulatory mechanisms governing the formation and retention of key volatile compounds remain unclear, warranting further exploration of metabolic pathways and enzymatic activities under non-thermal processing. In terms of fermentation, while *Saccharomyces cerevisiae* Aroma White demonstrated robust performance, alternative yeast strains (e.g., *Torulaspora delbrueckii*) or wild fermentations could enhance flavor complexity. Single-factor optimization of ultrasound parameters limited the understanding of synergistic approaches, such as ultrasound-enzyme hydrolysis or microbial co-fermentation, which could further boost bioactive compound yields. HS-SPME-GC–MS effectively profiled volatiles, but complementary techniques (e.g., GC × GC-TOF-MS, PTR-MS) are needed to capture a broader spectrum. Similarly, colorimetric assays for phenolics and flavonoids, though practical, lack specificity, and should be complemented by UPLC-MS/MS or HPLC-HRMS for precise quantification. Finally, while short-term sensory improvements were evident, the long-term stability of ultrasonic-induced changes remains uncharacterized, requiring accelerated shelf-life studies. Future research should focus on consumer feedback, process optimization, economic and environmental assessments, bioavailability, predictive modeling, and mechanistic insights to enhance the scientific and industrial relevance of non-thermal processing technologies in astragalus mead production.

## 5 Conclusion

This study explored the impact of high hydrostatic pressure, ultrasound, microwave irradiation, and natural aging on the physicochemical parameters, antioxidant capacity, color, and aroma profile of astragalus mead. Using single factor and RSM, optimal fermentation conditions for maximum flavonoid content and sensory quality were determined: impregnation for 12 h at 10 °C and fermentation at 20 °C. Among the treatments, ultrasonic processing was most effective, significantly increasing TPC (7.22 %) and TFC (9.41 %) compared to untreated wine. It also enhanced antioxidant activity, including ferric reducing power, hydroxyl radical scavenging, and total antioxidant capacity. Volatile compound analysis showed a 191.30 % increase in ester content from ultrasound treatment, contributing to floral and fruity notes, as confirmed by QDA. PLS-DA and WGCNA revealed distinct volatile profiles in the ultrasonic-treated mead, with ethyl caprylate as a key aroma differentiation compound. The turquoise module of WGCNA highlighted compounds associated with desirable sensory characteristics, such as floral and fruity notes. Ultrasonic processing outperformed traditional aging, microwave irradiation, and high hyperstatic pressure methods in enhancing both the nutritional and sensory attributes of astragalus mead. This study provides a foundation for developing efficient, non-thermal processing techniques to improve astragalus mead quality while reducing production time. Future research should refine ultrasonic parameters, assess bioavailability impacts, and explore large-scale consumer acceptance for industrial applications.

## CRediT authorship contribution statement

**Jianfeng Wang:** Writing – review & editing, Writing – original draft, Visualization, Resources, Data curation. **Xiangjin Kong:** Visualization, Methodology, Data curation, Conceptualization. **Yuqi Han:** Writing – review & editing, Investigation, Formal analysis. **Faisal Eudes Sam:** Visualization, Validation, Software. **Jixin Li:** Writing – review & editing. **Zhengmei Qi:** Validation, Software. **Yumei Jiang:** Writing – review & editing, Supervision, Resources, Project administration, Funding acquisition, Data curation.

## Declaration of competing interest

The authors declare that they have no known competing financial interests or personal relationships that could have appeared to influence the work reported in this paper.
