# Supplementary material for: Ultrasonic replacement of natural aging: Potential strategies for improving the color, antioxidant activity, and volatile compound profile of astragalus mead
Source: Ultrason Sonochem. 2025 Mar 20;116:107319. doi: 10.1016/j.ultsonch.2025.107319 (PMC11981771; doi:10.1016/j.ultsonch.2025.107319)

**Supplementary material**

**Ultrasonic replacement of natural aging: Potential strategies for improving the color, antioxidant activity, and volatile compound profile of astragalus mead**

Jianfeng Wang ^a,b,1^, Xiangjin Kong ^a,1^, Yuqi Han ^a^, Faisal Eudes Sam ^c^, Jixin Li ^a^, Zhengmei Qi ^d^, Yumei Jiang ^a,^**^*^**

**^a^** College of Food Science and Engineering, Gansu Agricultural University, Lanzhou 730070, China

**^b^** Key Laboratory of Geriatric Nutrition and Health, Ministry of Education, Beijing Technology & Business University, Beijing 100048, China

**^c^** College of Enology, Northwest A&F University, Yangling, Shaanxi 712100, China

**^d^** Gansu Runfengyuan Agricultural and Animal Husbandry Ecological Technology Co., Ltd., Yongdeng, Gansu 730300, China

*****Corresponding Author: Yumei Jiang E-mail: jym316@126.com

**Table S1.** Experimental values and coded levels of the independent variables used for the Box-Behnken design.

| Factor levels | Independent variable | | |
| --- | --- | --- | --- |
|  | Maceration time/h (A) | Maceration temperature/℃ (B) | Fermentation temperature/℃ (C) |
| -1 | 8 | 5 | 18 |
| 0 | 12 | 10 | 21 |
| 1 | 16 | 15 | 24 |

**Table S2.** Grading criteria for sensory evaluation score

| Item | Scoring criteria | Score |
| --- | --- | --- |
| Texture | Large amounts of suspended matter or sediment, which cannot be eliminated even by shaking | <10 |
|  | A small amount of suspension or precipitation is eliminated by oscillation | 10-14 |
|  | Clarified and homogeneous, without suspension or precipitation | 15-20 |
| Aroma | The odor is noticeably unbalanced and unpleasant | <20 |
|  | Astragalus, honey, sea buckthorn single odor prominence | 20-24 |
|  | The moderate astragalus medicinal flavor, the sweet honey aroma and the sweet and sour of sea buckthorn have a nice balance | 25-30 |
| Taste | Poor taste, too acidic, too sweet or accompanied by rancid flavors | <20 |
|  | Slightly less palatable, sweet or sour, no off-flavors in the mouth | 20-24 |
|  | Soft mouthfeel and suitable acidity and sweetness, no off-flavor | 25-30 |
| Color | Very dark color | <10 |
|  | Darker, pale-yellow color | 10-14 |
|  | Bright, light-yellow color | 15-20 |

**Table S3.** Grading criteria for quantitative descriptive analysis score of different astragalus wines.

| Aroma attributes | Definition and description of aroma attributes | Scoring criteria | Score |
| --- | --- | --- | --- |
| Floral | Associated with different flowers | No floral flavor | 1 |
|  |  | Weak floral flavor | 2-4 |
|  |  | Moderate floral flavor | 5 |
|  |  | Strong floral flavor | 6-8 |
|  |  | Very floral | 9 |
| Fruity | Associated with different fruits: acid, ripe and tropical | No fruity flavor | 1 |
|  |  | Weak fruity flavor | 2-4 |
|  |  | Moderate fruity flavor | 5 |
|  |  | Strong fruity flavor | 6-8 |
|  |  | Very fruity | 9 |
| Vegetal | Associated with gardens, green notes, dry leaves, and wood | No vegetal flavor | 1 |
|  |  | Weak vegetal flavor | 2-4 |
|  |  | Moderate vegetal flavor | 5 |
|  |  | Strong vegetal flavor | 6-8 |
|  |  | Very vegetal | 9 |
| Woody | Associated with oak-aging notes, characterized by aromatic compounds such as vanillin, guaiacol, and whisky lactones, contributing to the perception of vanilla, spice, coconut, and charred wood. It is influenced by the type and toasting level of the oak barrels used in aging | Very poor woody | 1 |
|  |  | Poor woody | 2-4 |
|  |  | Moderate woody | 5 |
|  |  | Strong woody | 6-8 |
|  |  | Very strong woody | 9 |
| Chemical | Not associated with food, it is characterized by its aggressiveness (smoked, phenolic, sulfuric, vinegary). | No chemical flavor | 1 |
|  |  | Weak chemical flavor | 2-4 |
|  |  | Moderate chemical flavor | 5 |
|  |  | Strong chemical flavor | 6-8 |
|  |  | Very chemical | 9 |
| Fresh | Sensation of freshness in the oral cavity (similar to that produced by mint) | No fresh flavor | 1 |
|  |  | Weak fresh flavor | 2-4 |
|  |  | Moderate fresh flavor | 5 |
|  |  | Strong fresh flavor | 6-8 |
|  |  | Very fresh | 9 |

**Table S4.** The design and results of RSM optimization.

| Test number | Impregnation time  (A, h) | Impregnation temperature  (B, ℃) | Fermentation temperature  (C, °C) | Overall score  (Y) |
| --- | --- | --- | --- | --- |
| 1 | 16.00 | 15.00 | 21.00 | 83.23 |
| 2 | 16.00 | 5.00 | 21.00 | 75.12 |
| 3 | 8.00 | 10.00 | 18.00 | 76.83 |
| 4 | 16.00 | 10.00 | 24.00 | 79.50 |
| 5 | 12.00 | 15.00 | 24.00 | 80.03 |
| 6 | 8.00 | 15.00 | 21.00 | 75.64 |
| 7 | 16.00 | 10.00 | 18.00 | 87.22 |
| 8 | 12.00 | 5.00 | 24.00 | 79.75 |
| 9 | 12.00 | 10.00 | 21.00 | 97.27 |
| 10 | 8.00 | 5.00 | 21.00 | 66.69 |
| 11 | 12.00 | 10.00 | 21.00 | 97.42 |
| 12 | 8.00 | 10.00 | 24.00 | 73.13 |
| 13 | 12.00 | 15.00 | 18.00 | 90.31 |
| 14 | 12.00 | 10.00 | 21.00 | 95.41 |
| 15 | 12.00 | 5.00 | 18.00 | 84.51 |
| 16 | 12.00 | 10.00 | 21.00 | 95.13 |
| 17 | 12.00 | 10.00 | 21.00 | 96.85 |

**Table S5.** Analysis of regression model variance and significance test results.

| Source | Sum of squares | Degree of freedom | Mean square deviation | *F*-value | *P*-value | Significance |
| --- | --- | --- | --- | --- | --- | --- |
| Model | 1474.92 | 9 | 163.88 | 53.69 | <0.0001 | ****** |
| A | 134.32 | 1 | 134.32 | 44.00 | 0.0003 | ****** |
| B | 66.93 | 1 | 66.93 | 21.93 | 0.0023 | ****** |
| C | 87.52 | 1 | 87.52 | 28.67 | 0.0011 | ****** |
| AB | 0.18 | 1 | 0.18 | 0.058 | 0.8169 | NS |
| AC | 4.04 | 1 | 4.04 | 1.32 | 0.2877 | NS |
| BC | 7.62 | 1 | 7.62 | 2.50 | 0.1582 | NS |
| A^2^ | 696.66 | 1 | 696.66 | 228.22 | <0.0001 | ****** |
| B^2^ | 295.89 | 1 | 295.89 | 96.93 | <0.0001 | ****** |
| C^2^ | 80.89 | 1 | 80.89 | 26.50 | 0.0013 | ****** |
| Residual | 21.37 | 7 | 3.05 |  |  |  |
| Lack of fit | 16.78 | 3 | 5.59 | 4.87 | 0.0801 | NS |
| Pure error | 4.59 | 4 | 1.15 |  |  |  |
| Cor. total | 1496.29 | 16 |  |  |  |  |

***** Significant difference (*p* < 0.05).

****** Highly significant difference (*p* < 0.01).

NS, no significance.

**Table S6.** Detailed information on the 12 compounds contained in the turquoise module.

| CAS | Compound name | Threshold | Aroma descriptors |
| --- | --- | --- | --- |
| 106-30-9 | Ethyl heptanoate | 2.2 | Brandy, fruit, wine |
| 7789-92-6 | 1,1,3-Triethoxypropane | 0.5 | Green, banana, aldehydic, fatty, cheesy |
| 100-66-3 | Anisole | 152.5 | Phenolic, gasoline, ethereal, anise |
| 143-08-8 | 1-Nonanol | 45.5 | Fat, floral, green, oil |
| 97-62-1 | Ethyl isobutyrate | 0.02 | Sweet, ethereal, fruity, alcoholic, fusel, rummy |
| 97-87-0 | Butyl isobutyrate | 93.6 | Fruit |
| 32665-23-9 | Isovaleric acid isopropyl ester | 24.5 | Apple, pineapple, fruity, winey |
| 2445-67-2 | 2-Methyl-1-propyl 2-methylbutyrate | 43 | Sweet, fruity |
| 2050-01-3 | Isopentyl isobutyrate | 87 | Fruity, ethereal, tropical, green, grape, cherry unripe, banana, apple, cocoa |
| 103-45-7 | Phenethyl acetate | 249.59 | Flower, honey, rose |
| 103-38-8 | Benzyl isovalerate | 11.5 | Fruit |
| 71-23-8 | 1-Propanol | 8505.6 | Alcohol, candy, pungent |

**Fig. S1.** Single factor experiment of ultrasonic treatment conditions.

**
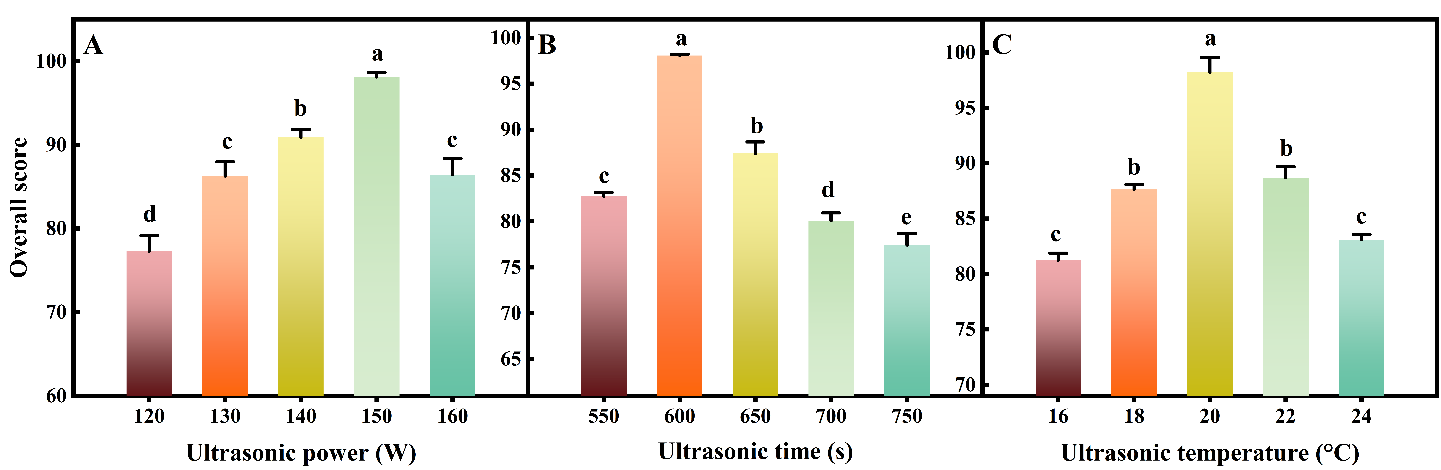
**

**Fig. S2.** Determination of the optimal soft threshold for weighted gene network co-expression analysis.


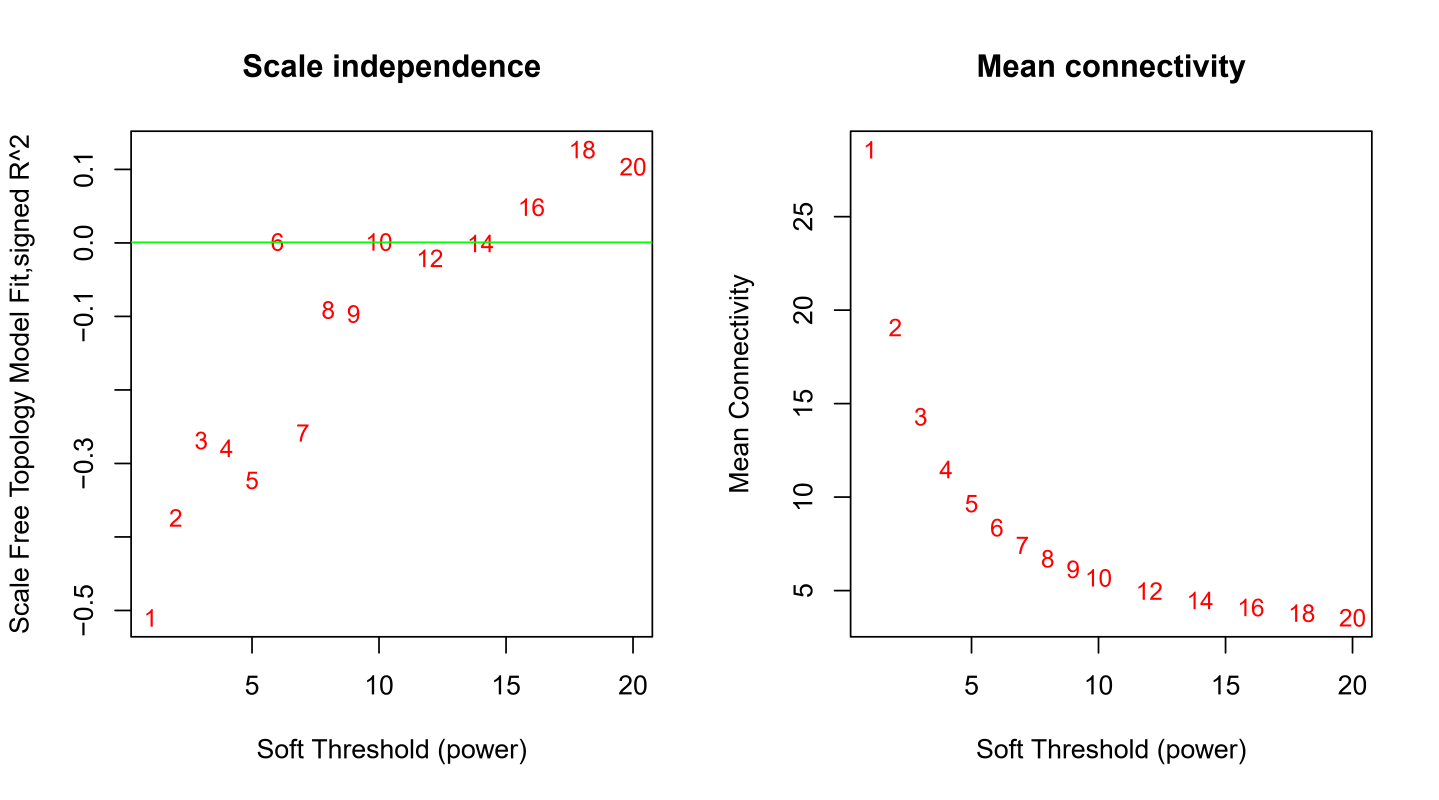

Supplement: Supplementary Data 1 [file mmc1.docx]
